# Associations between APOE Genotypes and Disease Susceptibility, Joint Damage and Lipid Levels in Patients with Rheumatoid Arthritis

**DOI:** 10.1371/journal.pone.0060970

**Published:** 2013-04-17

**Authors:** Marthe T. Maehlen, Sella A. Provan, Diederik P. C. de Rooy, Annette H. M. van der Helm - van Mil, Annemarie Krabben, Tore Saxne, Elisabet Lindqvist, Anne Grete Semb, Till Uhlig, Désirée van der Heijde, Inger Lise Mero, Inge C. Olsen, Tore K. Kvien, Benedicte A. Lie

**Affiliations:** 1 Department of Medical Genetics, University of Oslo and Oslo University Hospital, Ullevål, Oslo, Norway; 2 Department of Rheumatology, Diakonhjemmet Hospital, Oslo, Norway; 3 Department of Rheumatology, Leiden University Medical Centre, Leiden, The Netherlands; 4 Department of Rheumatology, Lund University, Lund, Sweden; 5 Department of Neurology, University of Oslo and Oslo University Hospital, Ullevål, Oslo, Norway; Harvard Medical School, United States of America

## Abstract

**Objective:**

Apolipoprotein E (*APOE*) genotypes are associated with cardiovascular disease (CVD) and lipid levels. In rheumatoid arthritis (RA), an association has been found with disease activity. We examined the associations between *APOE* genotypes and disease susceptibility and markers of disease severity in RA, including radiographic joint damage, inflammatory markers, lipid levels and cardiovascular markers.

**Method:**

A Norwegian cohort of 945 RA patients and 988 controls were genotyped for two *APOE* polymorphisms. We examined longitudinal associations between *APOE* genotypes and C-reactive protein (CRP), erythrocyte sedimentation rate (ESR) as well as hand radiographs (van der Heijde Sharp Score(SHS)) in 207 patients with 10 year longitudinal data. Lipid levels, cardiovascular markers and history of CVD were compared across genotypes in a cross sectional study of 136 patients. Longitudinal radiological data of cohorts from Lund and Leiden were available for replication. (N = 935, with 4799 radiographs).

**Results:**

In the Norwegian cohort, associations between APOE genotypes and total cholesterol (TC) and low-density lipoproteins (LDL) were observed (ε2<ε3/ε3<ε4, p = 0.03 and p = 0.02, respectively). No association was present for acute phase reactant or CVD markers, but a longitudinal linear association between *APOE* genotypes and radiographic joint damage was observed (p = 0.007). No association between *APOE* genotypes and the severity of joint destruction was observed in the Lund and Leiden cohorts, and a meta- analysis combining all data was negative.

**Conclusion:**

*APOE* genotypes are associated with lipid levels in patients with RA, and may contribute to dyslipidemia in some patients. *APOE* genotypes are not consistently associated with markers of inflammation or joint destruction in RA.

## Introduction

Disease severity varies between patients with rheumatoid arthritis (RA), and some studies have indicated that the severity of the disease is influenced by genetic factors [1], [2]. Patients with high disease activity over time are more likely to suffer from joint damage and increased disability as well as increased risk of cardiovascular disease (CVD) [3], [4], [5]. Identifying robust risk factors, which associate with disease severity have proven difficult in RA. Thus, increased knowledge of factors influencing the disease process is needed.

Apolipoprotein E (APOE) is a glycoprotein involved in lipid transport and metabolism. APOE binds to cholesterol or triglycerides and form a lipoprotein particle, which has a hydrophilic outer layer allowing it to be transported in the blood. APOE is important in the uptake of lipids as it mediates the binding between the lipoprotein particle and the low-density lipoprotein receptor [6]. There are different isoforms of APOE which are structurally and functionally different, and the APOE4 isoform is associated with higher lipid levels and increased risk of CVD in the general population [7]. There is increasing evidence that APOE may also play a role in immunomodulation and inflammation [8], [9], [10]. APOE has been shown to suppress proliferation of T-cells, modulate functions of macrophages as well as assist in the presentation of lipid antigens to immune cells [8], [9]. Genetic variants encoding the different APOE isoforms have been associated with a range of inflammatory and autoimmune diseases such as Alzheimer’s disease (AD) [11], psoriasis vulgaris [12]–[13], diabetic nephropathy [14], severity of hepatitis C infection [15], and levels of C-reactive protein (CRP) [16]. In a cross sectional study of patients with RA, *APOE* variants were associated with CRP, erythrocyte sedimentation rate (ESR) and Disease Activity Score (DAS28) levels indicating that APOE may play a role in severity of RA [17]. Moreover, Postigo et al recently investigated severity of collagen-induced arthritis (CIA) in an *ApoE (gene)* knockout mouse model [18]. They found that *ApoE* knockout mice developed an accelerated CIA, characterised by increased clinical severity, more radiographic damage, and higher expression of proinflammatory cytokines in the joints when compared with the CIA seen in the wild type control mice. Nonetheless the association between APOE and inflammation and radiological severity in RA is largely unknown.

There are three different isoforms of APOE, i.e.APOE2, APOE3 and APOE4, determined by two single nucleotide polymorphisms (SNPs) in the *APOE* gene (rs7412 and rs429358). These two SNPs give rise to three different alleles known as epsilon 2 (ε2), ε3 and ε4 [19]. A meta-analysis found a linear relationship between the *APOE* genotypes and both lipid levels and coronary risk, with ε2 carriers having lower coronary risk, total cholesterol (TC), low-density lipoproteins (LDL) and higher levels of high-density lipoprotein (HDL) compared with the most common genotype ε3/ε3, whilst ε4 carriers had higher coronary risk, higher TC and LDL levels but lower HDL levels than ε3/ε3 [7]. RA patients are known to have an altered lipid profile and to have increased mortality and morbidity from CVD which appears to be related to disease activity [20], [21]. Interestingly, the relationship between *APOE* genotypes and lipid levels has recently been found in an RA population [17], however, it is still unknown whether *APOE* genotypes are associated with CVD in patients with RA.

As recent literature suggests that *APOE* genotypes may be associated with disease susceptibility and/or severity in inflammatory diseases, we investigated their influence on RA severity and susceptibility. First, we further examined the association between *APOE* genotypes and lipid profile, and secondly explored whether *APOE* genotypes were associated with CVD related outcomes in a Norwegian RA population. We then went on to investigate the proposed relationship between *APOE* and inflammation in RA, by studying serum inflammatory markers as well as radiographic joint damage in the Norwegian EURIDISS cohort which had been followed longitudinally with repeated measures for 10 years [3], [22]. We tested our findings in two replication cohorts with longitudinal radiographic data from Sweden (Lund) and the Netherlands (Leiden).

## Materials and Methods

### Norwegian Patients and Controls

For the analyses on RA susceptibility, DNA was available from 945 Norwegian patients diagnosed with RA according to the 1987 American College of Rheumatology criteria [23] and 1026 Norwegian healthy controls. The patients were recruited from four separate Norwegian RA cohorts; 215 patients from the European Research on Incapacitating Disease and Social Support (EURIDISS) cohort, 615 patients from the Oslo RA register (ORAR), 81 from a cohort of early RA patients, and 34 from a cohort of patients starting tumour necrosis factor (TNF)-inhibitor therapy [22], [24]–[26]. Data on anti-citrullinated protein antibodies (ACPA) and rheumatoid factor (RF) were available for 885 and 887 patients, respectively. Clinical data mainly from the EURIDISS cohort was used to study RA severity (described below).

#### Cross sectional data – lipids and cardiovascular outcome measures

In 2007, 106 patients from the EURIDISS cohort participated in a 15-year follow-up examination; in addition 34 patients from the ORAR participated in a 10-year follow-up. All 140 patients (106 from EURIDISS and 34 from ORAR) had long standing disease in 2007 with a mean disease duration of 17.3 (95% CI 17.0–17.7) years) at time of examination. Patients from the EURIDISS cohorts had longer disease duration compared with patients from the ORAR cohort, (EURIDISS, 18.2 years (95% CI 17.9–18.5), ORAR, 14.7 years (95% CI 14.4–15.0)). The cardiovascular examination included details on cardiovascular risk factors, non-invasive measurements of intima media thickness (IMT) and examination of atherosclerotic plaques of the common carotid arteries [5], [27]–[28]. Ultrasonography was carried out by a single experienced sonographer on bilateral common carotid arteries. The images were later read by two experienced vascular physicians, and the intraclass correlation coefficient was above 0.95. Serum was analysed for TC, LDL, HDL and triglycerides along with several other biomarkers. Data collected at the follow-up in 2007 were used in the cross sectional analyses of associations between genotypes and lipid levels, carotid IMT, presence of plaques, and history of CVD events.

#### Longitudinal data – Radiographic progression and acute phase reactants

The 215 patients from the EURIDISS cohort had been followed longitudinally with hand radiographs, clinical assessments, and blood samples at baseline (1992) and after 1, 2, 5 and 10 years. All patients had baseline disease duration of ≤4 years and 54% were treated with synthetic disease modifying antirheumatic drugs (DMARDs) at baseline. The radiographs were scored by a trained reader with known time order, according to the Sharp van der Heijde method (SHS) [29]. Values of CRP, ESR, RF (IgM) and ACPA were available at the different time points. The methods used in assessing the soluble biomarkers and the scoring of the radiographs have been described previously [3], [22]. In the longitudinal analyses patients with ε2/ε4 genotype (n = 8) were excluded (see statistics for further explanation). Followingly, 207 patients were included in the longitudinal analyses of CRP or ESR. When studying radiographic damage, 176 patients had at least one radiograph and were eligible for the analyses. A total of 744 radiographs were included in the analyses.

### Replication Cohort - Lund

183 patients diagnosed with RA between 1985–1989 were included and followed prospectively for 5 years with annual radiographs of hands and feet [30]. Patients were initially diagnosed according to the ARA 1958 criteria, and all patients had less than 2 years disease duration at inclusion. 145 patient had both radiographs and DNA available, but patients with ε2/ε4 genotype (N = 7) were excluded. Therefore, 733 radiographs were available from 138 patients for the replication analysis. All radiographs were scored according to Larsen by one of two readers as described previously [30]. 44% of patients received DMARD treatment within the first year of follow up.

### Replication Cohort - Leiden

640 RA patients from the Leiden Early Arthritis Clinic Cohort had both DNA for SNP genotyping and radiographic data available. Patients with ε2/ε4 genotype (N = 19) were excluded, therefore 621 patients with a total of 3322 radiographs were included in the replication analysis. Patients were included at the time of diagnosis during the period 1993–2006, and were followed yearly for 7 years with radiographs of hands and feet. All x-rays had been scored using the SHS as previously described [31]. The treatment given during the study time varied according to year of inclusion but could broadly be divided into three treatment periods; non-steroidal anti-inflammatory drugs (1993–1995), chloroquine or sulphasalazine (1996–1998) and methotrexate or sulphasalazine (1999 onwards).

### Genotyping

Norwegian patients and controls, and patients from the Lund and Leiden cohort were genotyped for rs7412 and rs429358. *APOE* alleles were determined based on the haplotype conformation of the two SNPs as previously described [19]. In all datasets, Taqman technology (Applied Biosystems) was used for genotyping. In the Norwegian dataset 94 controls were genotyped twice for quality control. Error rate for the Norwegian as well as the Lund and Leiden cohorts was 0%. Genotyping success rate was above 98% for both SNPs in all cohorts. Genotypes were in Hardy Weinberg equilibrium (HWE) with a p-value above 0.05 for both patients and controls.

### Ethics Statement

The data inspectorate and the Regional Committees for Research Ethics in Eastern and Southern Norway had approved the original data collections. All patients in all three cohorts (Norway, Lund and Leiden) had given written informed consent before participation.

### Statistics

Chi square tests were used when testing for deviation in frequencies of genotypes (ε2/ε2, ε2/ε3, ε2/ε4, ε3/ε3, ε3/ε4 and ε4/ε4) or alleles (ε2, ε3 or ε4) between patients and controls. Association analyses were also carried out in the subsets of patients who were positive or negative for ACPA or RF, respectively.

The patients were divided into three genotype groups (ε2 carriers (ε2/ε2, ε2/ε3), ε3/ε3 or ε4 carriers (ε4/ε3, ε4/ε4)) when testing for associations with clinical and laboratory measures. The rationale behind this grouping was based on previous studies demonstrating that ε4 carriers have an increased CVD risk compared with the most common genotype ε3/ε3, whilst ε2 carriers have a decreased CVD risk compared with ε3/ε3 [7]. As the effects of ε2 and ε4 have been suggested to go in opposite directions, patients with ε2/ε4 genotypes were excluded from the analyses [7].

Demographic and baseline variables were compared between genotype groups using analysis of variance (ANOVA) for continuous variables and Chi-square for dichotomous variables. ANCOVA was used when comparing lipid levels and carotid IMT across genotype groups with adjustments for age, sex, and use of statins. A composite cardiovascular endpoint (presence of plaques or a past history of a cardiovascular event (defined as angina, stroke, previous coronary artery bypass graft, previous percutaneous coronary intervention/coronary stenting, myocardial infarction, and/or peripheral artery disease)) was compared between groups using Chi-square test for the univariate analyses and logistic regression for multivariate analyses adjusting for age, sex and statin use.

#### Longitudinal analyses

In the EURIDISS cohort we used linear mixed modelling analyses to investigate the longitudinal associations between *APOE* genotype group and repeated measures of CRP, ESR and radiographic SHS score [31]. SHS, CRP and ESR were log-transformed and each tested as dependent variables. Linear mixed modelling allowed us to use data from all time points per patient, with a maximum of five measurements per individual (baseline, 1, 2, 5 and 10 year follow-up). This model adjusted both for within-patient correlation and missing data. Based on previous studies, there was suggestive evidence of a linear relationship between *APOE* genotype group (ε2, ε3/ε3 and ε4) and markers of disease severity [17]. However, the literature is limited and therefore we did an initial assessment where we made no assumptions of the direction or size of effect of *APOE* genotype groups. This was done by examining *APOE* genotype group as a categorical factor, where ε3/ε3 was set as the reference group. Our data suggested a linear relationship between *APOE* and SHS, but not for CRP or ESR. Consequently, we proceeded to model *APOE* as a linear covariate where ε2, ε3/ε3 and ε4 were coded as 0, 1 and 2, respectively. An unstructured covariance matrix model fitted the data best according to Akaike’s information criterion [32]. The multivariate linear mixed models were adjusted for time, age, sex, ACPA status, disease duration at baseline and treatment (DMARD ever/never). When examining the effect on radiographic joint damage, adjustment for CRP was also included. The difference in the rate of joint progression over time (i.e. the slope of the curve) between the three *APOE* genotype groups was assessed by including an *APOE* x time interaction term. We adjusted for multiple testing when appropriate, using either Dunnets-Hsu or Sidaks method [33]–[34].

#### Replication analyses

The two replication cohorts from Lund and Leiden had repeated measures of radiographic progression. Hence, mixed modelling analyses with *APOE* as a linear covariate were carried out in the two replication cohorts [35]. Adjustments were made for age, gender, time and treatment. For the Lund cohort a dichotomous DMARD ever or never variable was available, while in the Leiden cohort, *year of inclusion* was used as a proxy for treatment as described in detail previously [35]. Fixed meta-analyses based on data from all three cohorts were carried out [36]. The power of detecting a statistically significant association between *APOE* and radiographic damage in the replication cohorts was based on a simplistic two-sample t-test between ε2 carriers and ε4 carriers with group difference from the longitudinal analysis and standard deviation from the baseline assessment.

Statistical analyses were carried out using SPSS 17.0 (SPSS, Chicago, IL, USA), Plink v1.07, SAS v.9.2 and Power and Sample Size Calculations v.3.0.43 [37]–[38].

## Results

### RA Susceptibility

No association was observed between *APOE* alleles or genotypes and RA susceptibility in the Norwegian cohort, as the frequency distributions were similar between the 945 patients and 988 controls (p = 0.96, Table 1). Patients were also stratified according to presence or absence of ACPA and RF, respectively. No associations were seen to these disease subsets (Table 1).

**Table 1:** *APOE* genotypes in RA patients and controls.

	Total	ε2/ε2	ε2/ε3	ε2/ε4	ε3/ε3	ε3/ε4	ε4/ε4	p
Controls (%)	988	8 (0.8)	105 (10.6)	34 (3.4)	551 (55.8)	254 (25.7)	36 (3.6)	
Patients (%)	945	7 (0.7)	109 (11.5)	30 (3.2)	512 (54.2)	254 (26.9)	33 (3.5)	0.96
ACPA +	544	4	66	17	286	150	21	0.85
ACPA −	341	3	37	10	195	84	12	0.99
RF +	477	3	59	16	254	128	17	0.91
RF −	410	4	46	9	223	112	16	0.84
Odds ratio (95% CI)		0.94 (0.34–2.62)	1.12 (0.83–1.50)	0.95(0.57–1.57)	1	1.08 (0.87–1.33)	0.99 (0.61–1.61)	

ACPA; anti-citrullinated protein antibodies, RF; rheumatoid factor. Frequencies of genotypes were compared between all patients or subsets of patients (i.e. ACPA positive patients) and controls using chi square test.

* Odds ratios were calculated using ε3/ε3 as the reference group.

### Cross Sectional Analyses – Lipids and Cardiovascular Outcome Measures

For the cross-sectional analyses of lipid levels, 136 Norwegian patients were available for analyses. Demographics and patient characteristics were similar across the patient groups (ε2 carrier, ε3/ε3 and ε4 carrier), though presence of RF was borderline significant (Table 2).

**Table 2:** Demographics and clinical measures of the 136 patients included in the lipid and CVD analyses.

	ε2 carrier	ε3/ε3	ε4 carrier	Overall p*
	n = 15¤	n = 81	n = 40	
Age at follow-up (sd)	62.3 (9.64)	62.1(12.3)	62.7(12.6)	0.96
Disease duration (sd)	16.7 (2.49)	17.5 (1.94)	17.4(1.90)	0.44
Gender [%]	13 [86.7]	65 [80.2]	27 [67.5]	0.19
ACPA positive [%]	8 [61.5]	40 [52.6]	20 [52.6]	0.83
RF positive [%]	9 [69.2]	46 [60.5]	15 [39.5]	0.06
CDAI (sd)	8.70 (6.30)	6.92 (7.38)	6.16 (7.07)	0.51
DAS28 (sd)	2.98 (0.82)	2.64(1.05)	2.49(1.01)	0.29
HAQ (sd)	0.68 (0.62)	0.73 (0.56)	0.72 (0.63)	0.95
Statins [%]	2 [13.3]	13 [16.3]	11 [27.5]	0.28
User of sDMARD [%]	10 [66.7]	55 [68.8]	25 [62.5]	0.79
User of bDMARD [%]	4 [26.7]	18 [22.2]	7 [18.4]	0.79

sd; standard deviation, Disease duration; disease duration in years at follow-up in 2007, ACPA; anti-citrullinated protein antibodies, RF; rheumatoid factor, CDAI; Clinical Disease Activity Index, DAS28; Diseases Activity Score 28, HAQ; Health Assessment Questionnaire, Statins; lipid lowering medications, sDMARD and bDMARD; synthetic and biologic disease-modifying antirheumatic drug,

* The three genotype groups (ε2carriers, ε3/ε3, and ε4 carriers) were compared by ANOVA or chi square test.

¤ Not all patients had data available for all analyses, but the maximum number of patients in each group was ε2 n = 15, ε3/ε3 n = 81, ε4 n = 40. Patients with ε2/ε4 gentoype were excluded (n = 4).

Levels of TC and LDL differed across the three *APOE* genotype groups (TC p = 0.03, LDL p = 0.02, Table 3). ε4 carriers had higher levels than the common variant ε3/ε3, and ε2 carriers had the lowest levels. This association was stronger when age, gender and the use of statin were included in the multivariate analyses. No association was seen between *APOE* alleles and levels of HDL or triglycerides (Table 3).

**Table 3:** Lipid levels and CVD outcome in RA patients across different *APOE* genotype groups.

	*APOE* genotype group			Univariate			Multivariate		
	ε2 carrier	ε3/ε3 carrier	ε4 carrier	Over all	ε2 vs. ε3/ε3	ε4 vs. ε3/ε3	Over all	ε2 vs. ε3/ε3	ε4 vs. ε3/ε3
	n = 15	n = 81	n = 40	p	p*	p*	p	p*	p*
TC	5.02 (1.33)	5.68 (1.08)	6.06 (1.52)	0.03	0.13	0.22	0.001	0.019	0.040
LDL	2.77 (1.24)	3.32 (0.87)	3.69 (1.31)	0.02	0.14	0.15	0.002	0.047	0.040
HDL	1.63 (0.49)	1.79 (0.57)	1.88 (0.65)	0.37	–	–	0.09	–	–
Triglycerides	1.40 (0.74)	1.24 (0.77)	1.27 (0.58)	0.75	–	–	0.67	–	–
IMT	0.77 (0.18)	0.78 (0.19)	0.75 (0.17)	0.66	–	–	0.14	–	–
Plaques	7 [46.7]	43 [55.1]	27 [67.5]	0.28	–	–	0.29	–	–
CVD event	1 [6.7]	14 [17.3]	8 [20.0]	0.59$	–	–	0.62	–	–
CVD/plaque	7 [46.7]	49 [61.3]	27 [67.5]	0.36	–	–	0.38	–	–

LDL: low density lipoproteins, HDL; High density lipoproteins, IMT; intima-media thickness (mm), CVD event; cardiovascular disease event (see Methods sections). All lipids are measured in mmol/L. Lipid levels and IMT were compared using ANCOVA, and unadjusted mean values with standard deviation (sd) are given in column 2, 3 and 4. CVD outcomes, which included previous CVD events or carotid plaques present on ultrasound (or both) were compared using Chi-square test; and the number of individuals affected with percentages [%] are given in column 2, 3 and 4. Multivariate analyses were adjusted for age, sex and current statin use. Not all patients had data on all variables and patients with genotype ε2/ε4 (n = 4) were omitted from the analyses.

* p-values were corrected for multiple testing using Dunnets-Hsu adjustment.

$ Exact test.

We also investigated whether *APOE* was associated with cardiovascular disease in RA patients. We did not find an association with a composite cardiovascular endpoint (p = 0.59, Table 3). Nor did we find *APOE* to be associated with presence of plaques (p = 0.28, Table 3) or carotid IMT in neither univariate nor multivariate analyses.

### Longitudinal Analyses – Radiographic Progression and Acute Phase Reactants

Patients from the Norwegian EURIDISS cohort had data available allowing us to investigate the longitudinal associations between *APOE* (ε2 carriers, ε3/ε3 and ε4 carriers) and changes in radiographic score (SHS), as well as levels of CRP and ESR over 10 years. Demographic data and baseline clinical measures were compared across the three genotype groups and no significant differences were seen except for CRP (Table 4).

**Table 4:** Demographic and baseline clinical data on patients included in the Norwegian longitudinal analyses (EURIDISS cohort), grouped by APOE genotype.

	Total	ε2 carrier	ε3/ε3	ε4 carrier	
	n = 207	n = 21	n = 125	n = 61	P*
Women [% ]	155 [74.9]	16 [76.2]	98[78.4]	41 [67.2]	0.25
Age (sd)	51.5 (12.9)	50.4 (11.3)	52.4 (12.9)	49.8 (13.5)	0.40
Disease duration (sd)	2.24 (1.18)	2.31 (1.10)	2.23 (1.18)	2.24 (1.19)	0.96
ACPA positive [% ]£	126 [60.9]	15 [71.4]	75[60.0]	36 [59.0]	0.57
RF positive [% ]£	113 [54.6]	12 [57.1]	75 [60.0]	26 [42.6]	0.08
CRP (sd)	12.5 (17.3)	9.10 (10.7)	15.2 (19.9)	7.87 (11.7)	0.02
ESR (sd)	26.2 (20.2)	27.7(16.2)	27.6 (21.6)	22.8 (18.3)	0.29
HAQ (sd)	0.94 (0.64)	0.86 (0.56)	0.97 (0.66)	0.93 (0.65)	0.74
LnSHS(sd)	1.24 (1.27)	1.58 (1.49)	1.31 (1.24)	0.98 (1.25)	0.19
sDMARD users [% ]	112 [54.1]	13[61.9]	65 [52]	34 [55.7]	0.67

sd; standard deviation, Disease duration; disease duration at baseline in years, ACPA; anti-citrullinated protein antibodies, RF; rheumatoid factor, CRP; C-reactive protein (mg/L), ESR; erythrocyte sedimentation rate (mm/hour), HAQ; Health Assessment Questionnaire, LnSHS; log transformed Sharp van der Heijde score, sDMARD; synthetic disease-modifying anti rheumatic drugs.

* The three genotype groups (ε2 carriers, ε3/ε3, and ε4 carriers) were compared by ANOVA or Chi-square test.

£ For RF and ACPA, patients were viewed as positive if they had at least one positive test during the 10 year follow-up.

In the longitudinal analyses a significant linear association was seen for the three *APOE* genotype groups and radiographic score (*APOE* linear, p = 0.007, Table 5; model A). These results suggested that carriers of the ε2 allele had the highest radiographic score, followed by ε3/ε3, and with ε4 carriers having the least damage (ε2 carriers >ε3/ε3>ε4 carriers). This significant association was maintained after adjustments for time, CRP, DMARD treatment, ACPA status, age and gender (p = 0.01, Table 5; model B).

**Table 5:** Longitudinal analyses of *APOE* genotype and radiographic score, CRP and ESR as dependent variables in the EURIDISS cohort.

		Categorical£				Linear$	
		ε2 carrier		ε4 carrier		APOE linear	
Model¥	P§	Coefficient (95% CI)	p*	Coefficient (95% CI)	p*	Coefficient (95% CI)	p
**SHS**							
a	0.03	0.41 (−0.29 to 1.11)	0.34	−0.44 (−0.93 to −0.05)	0.09	−0.43 (−0.74 to −0.12)	0.007
b¤	0.04	0.27 (−0.35 to 0.89)	0.54	−0.41 (−0.86 to 0.04)	0.08	−0.36 (−0.64 to −0.08)	0.012
**CRP**							
a	0.13	0.07 (−0.30 to 0.43)	0.89	−0.20 (−0.44 to 0.04)	0.12	–	–
b	0.18	0.07 (−0.29 to 0.44)	0.89	−0.18 (−0.43 to 0.07)	0.19	–	–
**ESR**							
a	0.22	0.12 (−0.24 to 0.48)	0.72	−0.15 (−0.39 to 0.09)	0.31	–	–
b	0.50	0.12 (−0.24 to 0.47)	0.72	−0.08 (−0.32 to 0.17)	0.72	–	–

SHS, CRP and ESR have all been log-transformed.

¥ Models were adjusted for: a = time, b = time, age, sex, disease duration, ACPA and DMARD treatment (ever/never).

¤ Model B with SHS outcome was also adjusted for CRP. For the models investigating CRP or ESR n = 207, while for joint damage (SHS) n = 176. Patients with genotype ε2/ε4 were omitted from the analyses.

£ In the analyses where *APOE* genotype is a categorical factor, ε3/ε3 is the reference group.

$ In the linear analyses (*APOE* linear), ε2, ε3/ε3, and ε4 are coded 0, 1 and 2 respectively.

§ Overall p-value.

* P-values corrected for multiple testing using Sidaks’ adjustment.

**Figure 1:**
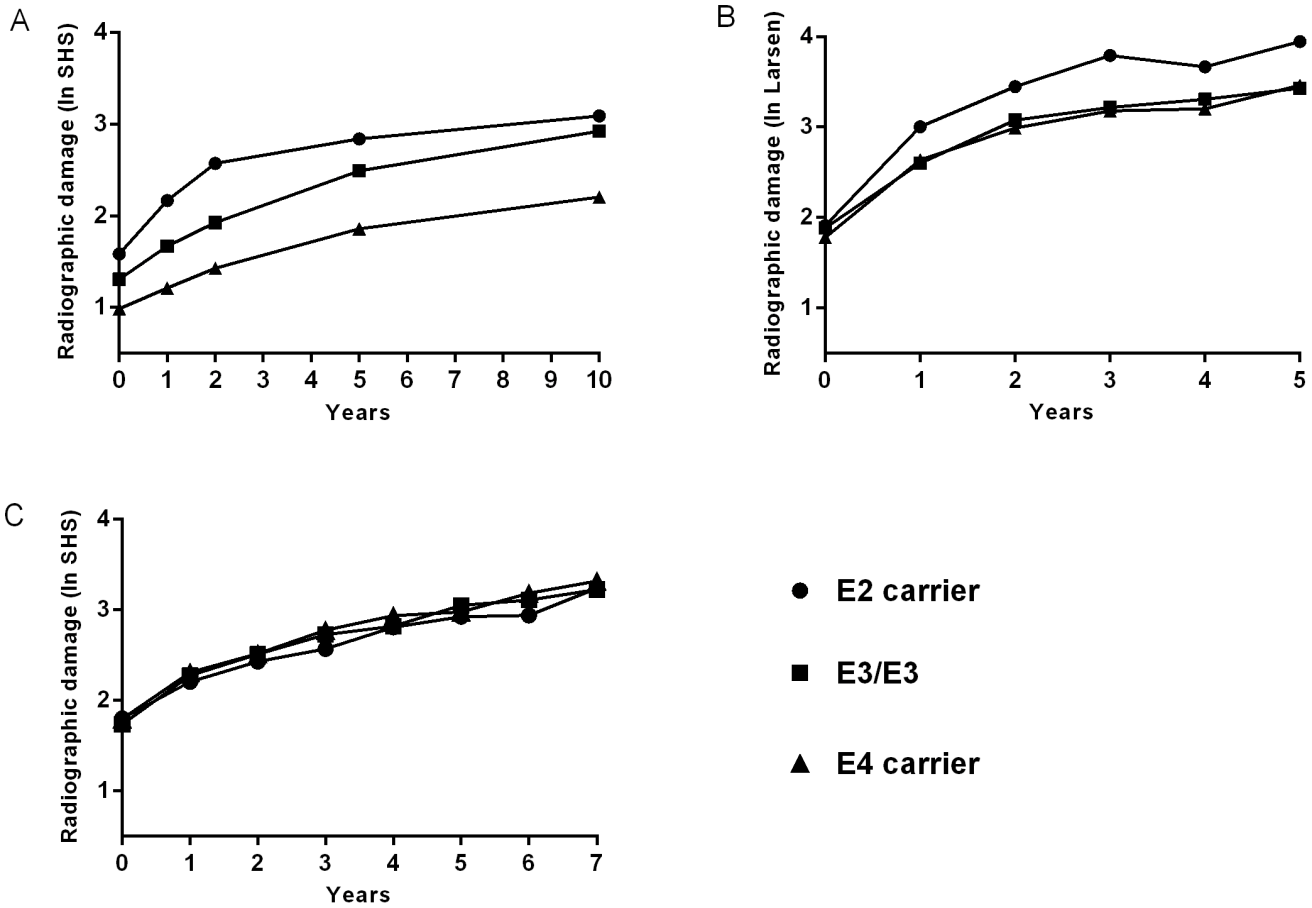
Mean radiographic joint damage in patients with RA in the three different cohorts grouped by *APOE* genotype. A = Norway, B = Lund (Sweden) and C = Leiden (Netherland). The Y-axes shows radiographic damage, either as log-transformed van der Heijde Sharp score or log transformed Larsen score (Lund). The x-axes show years of follow-up. Number of patients included: Norway (EURIDISS); n = 176, Leiden n = 621, Lund n = 138.

The association seen with radiographic damage was then evaluated in the Lund and Leiden cohorts. Based on the Norwegian estimate, the Lund and Leiden cohort had a power of 0.36 and 0.97, respectively to detect a statistical significant association between *APOE* and radiographic damage. However, neither cohort could replicate the association seen in the Norwegian cohort (Table 6). The mean radiographic score (log-transformed) for each year of follow-up was calculated for the three separate cohorts and are shown in Figure 1 (a, b and c). Figure 1 highlighted intriguing differences between the three cohorts. In the Norwegian EURIDISS cohort a difference in joint damage was visible at baseline and maintained over time. However as the lines representing the three *APOE* groups were parallel (Figure 1a), there was no evidence that *APOE* influenced the rate of progression over time (APOE*time, p = 0.96). In the Lund cohort, there was no difference between the groups at baseline but after 5 years follow-up the carriers of the ε2 allele had numerically the most radiographic damage (Figure 1b). No difference in SHS across the *APOE* groups was observed in the Leiden cohort neither at baseline nor after seven years, as demonstrated by the overlapping lines in Figure 1c. There was no statistically significant effect of *APOE* on rate of joint progression in either the Lund or the Leiden cohort (Lund, APOE*time p = 0.30, Leiden APOE*time p = 0.32). The fixed meta-analysis of *APOE* on radiographic damage gave no significant results (Table 6).

**Table 6:** Longitudinal analyses and meta-analyses for *APOE* and radiographic joint damage for 3 cohorts.

	Estimate (95% CI)	p
Norway *APOE*	−0.36 (−0.64 to −0.08)	0.01
Leiden *APOE*	0.03 (−0.08 to 0.13)	0.59
Lund *APOE*	−0.12 (−0.33 to 0. 09)	0.30
Meta-analysis		0.41

For *APOE*, the three genotype groups, ε2 carrier, ε3/ε3, and ε4 carrier are coded 0, 1 and 2 respectively. The coefficient estimates and 95% confidence interval (95% CI) from the longitudinal analyses give a measure of the difference in radiographic joint damage score of adjacent *APOE* genotype groups within each cohort. E.g. in the Norwegian cohort individuals with ε3/ε3 genotype had 0.36 lower radiographic joint damage score than individuals carrying ε2. The coefficient estimates are given on the natural log (ln) scale for radiographic joint damage score. Longitudinal analyses were adjusted for time, age, gender and treatment. Additional adjustments including presence of anti citrullinated protein antibodies and disease duration at baseline were included in the analyses of the Norwegian cohort. A fixed effect meta-analysis was carried out and the p-value is shown.

Levels of acute phase reactants were compared between genotype groups in the Norwegian cohort. Baseline CRP levels differed between genotype groups (Table 4), but this association was not confirmed in the longitudinal analyses (overall p = 0.13, Table 5), and the result did not change in the adjusted analyses. Nor did we find an association between genotype groups and ESR (Table 5).

## Discussion

We investigated the relationship between *APOE* genotypes and disease susceptibility, severity, lipid levels and CVD in an RA population. We replicated the association with TC and LDL levels reported from the general population. In contrast to the associations with lipid levels, we could not replicate the association seen with levels of inflammatory markers nor with disease severity as we found no evidence of association with joint damage in the overall meta-analyses [17].

There are several shared characteristics between atherosclerosis and inflammatory diseases such as RA. Elevated CRP is associated with disease severity and future cardiovascular events in both conditions, and smoking is a shared environmental risk factor [21], [39]–[40]. Furthermore, one could hypothesise that genetic risk factors are shared between the two diseases. APOE plays an important role in metabolism of triglyceride-rich lipoproteins, and *APOE* genotypes have been shown to be associated with changes in the lipid profile in the general population [7]. In this study, we replicated the association seen with TC cholesterol and LDL, which suggests that *APOE* may contribute to the development of dyslipidaemia in patients with RA [7], [17]. Furthermore, *APOE* has also been found to be associated with increased atherosclerosis and CVD [7], [41]. Increased IMT and presence of plaques are both indicative of atherosclerosis and are associated with increased risk of cardiovascular events. We could not find a statistically significant association between *APOE* and markers of cardiovascular disease. Still as the effect of *APOE* risk variants on CVD is small in the general population, our negative finding cannot rule out *APOE* as a risk factor for atherosclerosis and CVD in RA patients. Larger sample size will be needed to address this in future studies in RA.

*APOE* is not only an important risk factor for CVD but also late onset AD. Individuals who carry two copies of ε4 have 8 times increased risk of developing AD [11]. The current study including Norwegian RA patients and controls is the largest association study done on *APOE* and RA, and our findings did not indicate an increased risk of RA, and this finding is consistent with recent studies [17].

A key question in RA pathology is why joint damage varies between individuals. Known factors explain only 30% of the variance in joint damage [42]. In order to study predictors of disease severity, radiographic joint damage has been favoured as it is thought to reflect cumulative inflammation over time, and can therefore be considered as a more informative estimate of disease severity than a single measure of CRP or DAS28 [43]. In a cross sectional study of RA patients, Toms and colleagues found the ε4 allele was associated with lower, and ε2 allele with higher levels of ESR, CRP and DAS28 when compared with the common ε3/ε3 genotype [17]. In the current study, we tested this hypothesis in a longitudinal cohort with repeated measures, but could not replicate the association with CRP and ESR. Notably, we did find a similar relationship with radiographic joint damage (ε4<ε3/ε3<ε2) in our Norwegian exploratory cohort, but we were unable to replicate this finding.

In the current study, the number of patients in the replication cohorts was 4 times as high as in the Norwegian exploratory cohort, making a true relationship between *APOE* and joint damage in RA unlikely. Furthermore, the three cohorts used in this study were considered comparable as they were all initiated in a time when aggressive treatment was uncommon and with no or limited access to modern, biological DMARDs. In addition, all cohorts had a longitudinal design with repeated measurements over time making them better suited for identification of predictors of radiographic progression. Still, no cohorts are alike and there might exist considerable heterogeneity between the three cohorts with respect to access to treatment, frequency of follow-up, disease duration and other clinical characteristics which in turn might influence the outcome. In the Leiden cohort, 83% of patients had been treated with a DMARD within the first year compared with 54% and 44% of patients from Norway and Lund, respectively. Differences in type of DMARDs given in the three cohorts may differ, but these data are not available. Patients in the Lund and Leiden cohorts had shorter disease duration at baseline, emphasising that these patients started treatment earlier than the Norwegian patients. Percentages of ACPA and RF were comparable between the Norwegian and Dutch patients (59% and 53%) but higher for the Swedish patients (77.5%). We adjusted for possible confounders such as treatment, and although, there would undoubtedly still exist subtle differences between the cohorts which may have influenced joint progression, we find it unlikely that these differences can account for the negative results in the replication cohorts.

However a possible explanation for the contradicting findings could be population specific interactions and/or environmental factors. In a recent study published in Archives of Neurology, the authors investigated the association between carrier status of *APOE* ε4, exercise and amyloid deposition in healthy individuals [44]. Interestingly, the authors found an interaction between ε4 and exercise, suggesting that the effect of ε4 on amyloid deposition was modified depending on the amount of exercise an individual had been exposed to. This finding highlights the complexity of studying genetic risk factors, as there may be important interactions that are unaccounted for which at the end may influence the results. Although, our overall findings do not support a relationship between *APOE* and disease severity, we cannot rule out that *APOE* genotypes do affect the biological disease processes in RA. The true relationship may be far more complex.

Taken together, our study provides additional evidence supporting the influence of *APOE* genotypes on lipid levels in patients with RA, but no association was seen with CVD. In this study, with longitudinal radiographic data on more than 900 patients, we did not find consistent evidence supporting the proposed link between *APOE* genotypes and disease severity in RA.
